# Integrating Cognitive Radio with Unmanned Aerial Vehicles: An Overview

**DOI:** 10.3390/s21030830

**Published:** 2021-01-27

**Authors:** Guilherme Marcel Dias Santana, Rogers Silva de Cristo, Kalinka Regina Lucas Jaquie Castelo Branco

**Affiliations:** Institute of Mathematics and Computer Science, Universidade de São Paulo, São Carlos, SP 13566-590, Brazil; guilherme.santana@usp.br (G.M.D.S.); rogers.cristo@usp.br (R.S.d.C.)

**Keywords:** unmanned aerial vehicles, cognitive radio networks, software defined radio, network sensing, security, internet of flying things, machine learning, energy management

## Abstract

Unmanned Aerial Vehicles (UAVs) demand technologies so they can not only fly autonomously, but also communicate with base stations, flight controllers, computers, devices, or even other UAVs. Still, UAVs usually operate within unlicensed spectrum bands, competing against the increasing number of mobile devices and other wireless networks. Combining UAVs with Cognitive Radio (CR) may increase their general communication performance, thus allowing them to execute missions where the conventional UAVs face limitations. CR provides a smart wireless communication which, instead of using a transmission frequency defined in the hardware, uses software transmission. CR smartly uses free transmission channels and/or chooses them according to application’s requirements. Moreover, CR is considered a key enabler for deploying technologies that require high connectivity, such as Smart Cities, 5G, Internet of Things (IoT), and the Internet of Flying Things (IoFT). This paper presents an overview on the field of CR for UAV communications and its state-of-the-art, testbed alternatives for real data experiments, as well as specifications to build a simple and low-cost testbed, and indicates key opportunities and future challenges in the field.

## 1. Introduction

An Unmanned Aerial Vehicle (UAV), also known as drone, is an aircraft without passengers on board. Thus, the term “unmanned” implies a total absence of humans within the aircraft [1]. In this regard, UAVs need not only hardware and software capable of providing stability and preprogrammed flight navigation control [2], but also robust, effective, and secure communication technologies that enable them to communicate with base stations, air traffic controllers, other UAVs, or other devices and computers [1].

Although UAVs were initially designed for military action, the mass production of high-performance, low-cost, intelligent UAVs has made them suitable for different applications. These applications include video streaming, amateur photography and filming, people and environment monitoring, rescue and research, traffic control, and disaster recovery [3,4]. In fact, UAVs are currently being considered as an important part of the next generation of wireless networks 5G with the so-called cellular-connected UAVs [5], where they play a critical role to provide great performance improvements, thus bringing more opportunities to the Internet of Things (IoT) and more diversity for 5G communications.

Likewise, while performing their missions, the UAVs may work within an IoT [6] context when equipped with IoT devices. Herewith, the UAVs will form an innovative IoT platform operating in the skies, thus being part of a new concept known as the Internet of Flying Things (IoFT) [7]. For this purpose, UAVs need to support instantaneous and real-time communication, and offer access to high-resolution files (e.g., videos streaming and high-definition images), even if they are in an overcrowded area. That is the reason why 5G networks are considered a key enabler for the IoT and the IoFT. In this sense, the ultra-reliable and low latency (URLLC) mode of communication expected with 5G networks would not only provide the UAVs with the necessary IoT requirements but also provide coverage at high altitudes. Furthermore, the tasks of 5G networks may support remote change, and the planning of flight routes will be enhanced with the potential benefits of a 5G communication, thus preventing UAVs from colliding with each other [8].

Regarding the impact on spectral usage expected for the massive connections of UAVs in 5G, an important issue to be addressed is how these devices will be connected to future networks in a spectral efficient manner. It is known that despite the need for more robust and secure communication UAVs traditionally operate within unlicensed spectrum bands (i.e., open/free spectrum), such as IEEE S-Band, IEEE L-Band, and ISM, fixedly defined in their hardware. Therefore, the UAVs increasingly face competition, thus competing with a growing number of mobile devices (e.g., smartphones and tablets), operating within other wireless networks (e.g., Wi-Fi and bluetooth) that use the same spectrum bands. However, besides interfering the UAVs communications, this competition will not be bearable anymore, once a massive number of UAVs is expected to be connected, which will lead to a serious problem of spectrum scarcity and security-related problems [9]. In this context, Cognitive Radio (CR) emerges as a promising technology to solve these issues by enabling Dynamic Spectrum Access (DSA) [10].

Proposed by Mitola [11], CR is a smart wireless communication implemented in a Software-Defined Radio (SDR). An SDR sets its transmission frequency in software rather than in hardware, thus allowing the CR to intelligently switch to different channels. To switch channels, CR senses the radio spectrum environment around it and adapts its own configurations accordingly to increase reliability and efficient spectrum usage [10]. Furthermore, CR is considered a key enabling technology for 5G in emerging IoT applications [12]. Therefore, CR can provide UAVs with promising features for their massive deployment, such as reduced energy consumption and delay, opportunistic spectrum use based on application requirements, and a security enhancement, as CR does not suffer the effects of some conventional attacks.

These features would allow CR-based UAVs to perform in situations where traditional UAVs face limitations, or are often subject to being hacked. Moreover, CR alongside 5G would permit the UAVs to work in an IoFT role within an IoT context. This would fulfill the increasing growth of applications requiring highly connected devices, such as Smart Cities [13]. However, although UAVs and CR are well-established research fields, the integration between the two is fairly unexplored [9]. Many issues remain open, such as the impact of the UAVs mobility on CR and the definition of hardware compatible with both. These open issues allow new research opportunities and advances into the state-of-the-art.

In this paper, we provide a comprehensive overview of background on CR for UAV communication, and the ongoing research regarding the integration between the two. We also specify the steps to build a simple and low-cost CR-based UAV testbed. Finally, we present key opportunities and future challenges in the field.

## 2. Related Works

The first step towards the proper development of a CR-based UAV is choosing a suitable SDR-UAV combination. With a particular emphasis on SDR hardware and software that can be used for aerial wireless exploration and analysis, Powell et al. [14] provides a comparative overview of SDRs. They address specifications for SDR hardware, features of available SDR hardware that are acceptable for small UAVs, and measurements of power. They also present SDR software specifications, open-source SDR software available, and SDR software calibration/benchmarking. Finally, the authors present Aerial Experimentation and Research Platform for Advanced Wireless (AERPAW) as a case study, and address various different experiments that can be sponsored by SDRs on that platform to verify/test possible wireless advancements, protocols, and technologies.

CR aircraft applications are also the subject of recent surveys. Jacob et al. [15] carried out a survey on CR for aeronautical applications. Although their survey is not directly related to UAVs, they present valuable information about studies involving CR and UAS. Notwithstanding, Saleem et al. [9] undertook a survey on the integration of CR with UAVs. They discussed and highlighted a variety of challenges, issues, and future research in the field.

## 3. Background

Herein, we introduce some background information required to understand this work. First, we give an overview of UAVs. Second, we present a general description of the concepts related to CR and its operation.

### 3.1. Unmanned Aerial Vehicles

UAVs are described with different terms, such as drones and Unmanned Aerial Systems (UAS). These terms vary according to the research field, often describing the same thing though [1]. In this work, UAV is defined as an aerial vehicle, with an embedded computer platform, capable of flying with no human pilot on-board.

UAVs can be remotely controlled and/or autonomous, and they can perform a variety of tasks. Although initially purposed for military tasks, there is an increasing growth in civilian, commercial, and scientific applications (e.g., traffic surveillance, communication relays, disaster management, data and image acquisition, etc.) [16].

**Figure 1:**
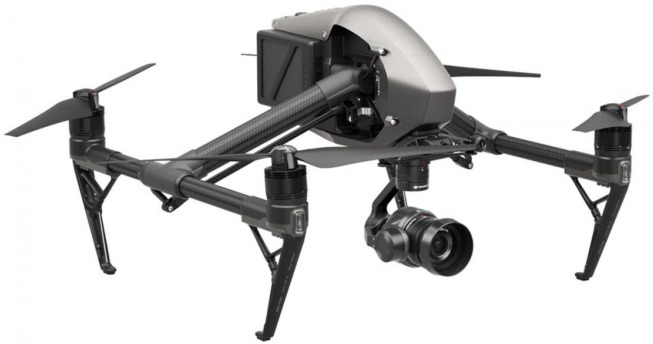
DJI Inspire 2, a mini UAV weighing around 3.3 kg and capable of a flight time up to 27 min [17].

As shown in Table 1, UAVs can be classified according to their weight, altitude and endurance [9]. Figure 1 shows an example of a mini UAV, the Inspire 2, by DJI, as categorized by Table 1 [17]. This UAV weighs around 3.3 kg and its flight reaches up to 27 min.

Although there is a wide variety of UAVs, this work focuses on their communication, considering micro and mini UAVs battery, space, and weight constraints.

For mini and micro UAVs, the battery represents an important portion of their weight. The DJI Inspire 2, for example, typically works with a pair of 4280 mAh batteries, weighing 515 g each. Although over 30% of this UAV weight is assigned to batteries, it is only able to fly 27 min per charge.

### 3.2. Cognitive Radio Networks

The European Telecommunications Standards Institute (ETSI) defines CR as a radio that can sense and understand the radio environment and policies, and monitor usage patterns and users’ needs; autonomously and dynamically adapts according to the radio environment, so that it can achieve predefined objectives, such as efficient utilization of spectrum; and learn from the environment and the results of its own actions, so it can further improve its performance [18].

CR is also able to operate as a secondary user (SU) within a spectrum band of a licensed user or primary user (PU). In [12,19], the Resolution ITU-R 58-2 of the International Telecommunications Union (ITU) states that “the introduction of CRSs in any radiocommunication service needs to ensure that coexistence within radiocommunication services and the protection of other radiocommunication services sharing the band and in the adjacent bands are maintained or improved” [20]. In practice, clearer parameters on how that action is done is usually defined by local regulatory agencies.

CR is described as an intelligent SDR with the following components:

1. spectrum sensing: identifies the available spectrum and detects PUs when operating in a licensed band,
2. spectrum management: selects the best available channel,
3. spectrum sharing: coordinates accessibility to the available channel with other users, and
4. spectrum mobility: vacates the channel when a PU arrives.

**Figure 2:**
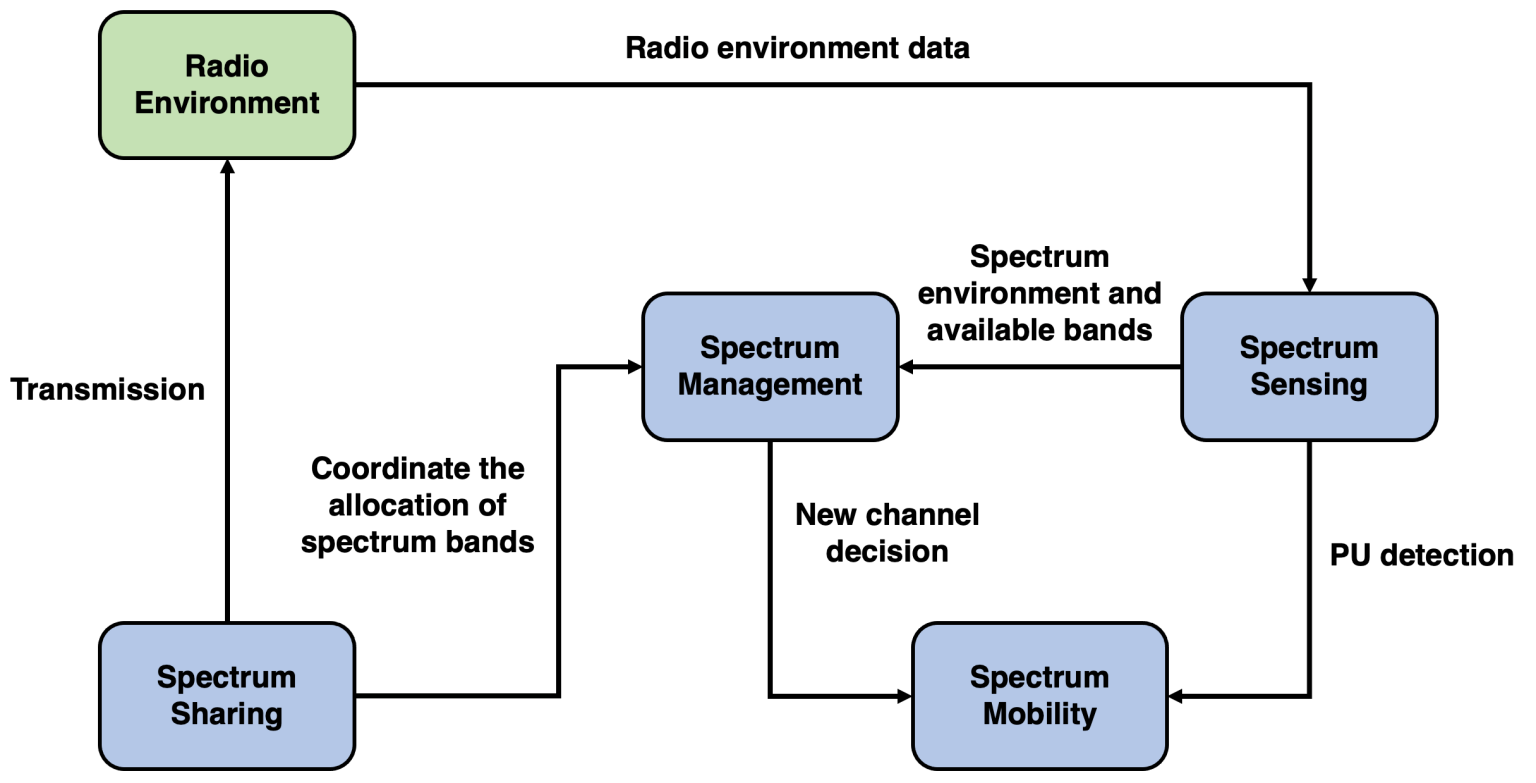
The interaction among CR components. Adapted from the work in [21].

Figure 2 shows the interaction among the CR components. First, the spectrum sensing component is responsible for collecting data from the radio environment, and it keeps sending the gathered radio data to the spectrum management component. When a PU is detected, it also notifies the spectrum mobility component, then a spectrum handover must be executed. The spectrum management component is responsible for submitting the channel with higher availability to the mobility component of the spectrum, because when the current channel needs to be vacated, a new channel is used. Finally, to organize the allocation of spectrum bands, the spectrum sharing portion is responsible for interacting with the radio environment.

In practice, CR consists of a hardware associated with an intelligent software. Usually, the hardware consists of a radio platform, generally an SDR, and a computational platform. Most computational platforms used for CR applications are single-board computers, such as the ODROID [22], the Raspberry Pi [23], and the BeagleBoard [24]. The Universal Software Radio Peripheral (USRP) from Ettus Research [25] and the Wireless Open-Access Research Platform (WARP) from Rice University are both SRDs and the most common radio platforms used in CR [26,27].

**Figure 3:**
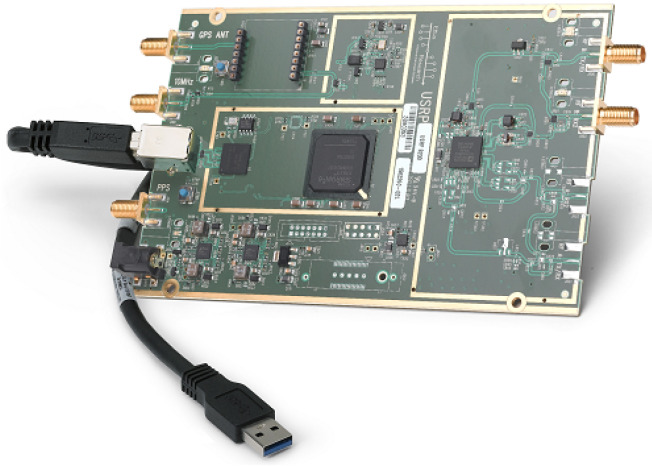
USRP B200 by Ettus Research. Its price is currently $905.00.

Figure 3 shows one of the most usual SDR hardware in research experiments: the USRP B200 by Ettus Research. It provides full-duplex wireless communication within a frequency range starting from 70 MHz varying up to 6 GHz. Its architecture offers compatibility with a variety of software and frameworks, such as C++, Python, GNU Radio, Amarisoft LTE 100, and OpenBTS.

## 4. CR-Based UAVs

A CR-based UAV may be seen as a UAV with an SDR platform embedded to it. It should also contain a computational unity that interacts with the SDR platform in order to autonomously take decisions regarding the radio spectrum usage. In this section, we first discuss the need for CR-based UAVs, potential applications, and hardware/software characteristics.

### 4.1. The Need for CR-Based UAVs

Herein, we define the most prominent aspects regarding the paramount importance of CR in the context of UAVs.

#### 4.1.1. Security

Communication security is critically important for UAVs. These aircraft are also considered critical systems, a security issue, which can be used to manage confidential information, thus, in a UAV, may often represent a serious safety issue. Some conventional attacks, such as jamming and location spoofing (sometimes referred to as GPS spoofing or GNSS spoofing), could lead the base station to lose the UAV. This problem is particularly evident in overcrowded or hostile areas.

Jamming is an attack in the physical layer which causes a high interference to a spectrum band by overloading it. It may provoke the attacked devices to present an excessive energy consumption due to package retransmission, or even interrupt its communication channel [7]. When a CR is under a jamming attack, it simply understands that spectrum band as being busy or overcrowded, and then it switches its transmission to a new channel, thus avoiding the attack. In order to succeed in their jamming attempt, the attackers would have to keep detecting the new CR frequency and switch to the same frequency. For this, however, the attackers themselves would need to also be equipped with a CR device and more sophisticated algorithms.

Location spoofing attacks also happen in the physical layer, and they have become more frequent. It happens when an attacker uses a signal that is stronger than and mimics the attributes of a genuine location satellite signal to spoof the receiver [7]. By using a location spoofing attack, attackers may capture UAVs and/or take control of their flight path. Such attacks have become easy to launch [28]. However, location spoofing attacks are much more complex to execute to CR devices. The work in [29] provides some strategies for spoofing attack detection and countermeasure solutions in CR networks.

Therefore, UAVs may benefit from CR technology security. Conventional jamming and GPS spoofing strategies may be easily avoided by CR. These attacks are only possible to CR when the attacker is also using a CR with different and specific strategies; therefore, attacking a CR device is a more complex problem than attacking conventional wireless devices. Herein, we define the most prominent aspects regarding the paramount importance of CR in the context of UAVs.

#### 4.1.2. Energy Efficiency

Although CR devices may represent a computational overload to UAVs, they may be capable of actually reducing energy consumption to these aircraft. Because the UAVs operate in overcrowded spectrum bands, they are susceptible to a high number of loss, thus increasing packet retransmission. The energy consumed for packet retransmission may be greatly reduced when a UAV is equipped with a CR.

Li et al. [30] proposed a method to maximize energy efficiency based on a joint optimization with medium access control (MAC) and physical layers, considering CR networks. In their scenario, a CR user senses different channels simultaneously and uses some idle ones for data transmission. The authors showed that the more channels the CR device is able to use, the more efficient is its bits/joule ratio throughput. The bits/second ratio also increased with the number of channels used.

#### 4.1.3. Spectrum Scarcity

Despite a massive growth in the number of wireless connected devices, most of the radio spectrum is underutilized. Because of the fixed spectrum allocation policy, a big portion of the radio spectrum is reserved to sporadic PUs, while other spectrum portions are overloaded, such as Wi-Fi and mobile bands. Moreover, the UAVs traditionally operate within unlicensed spectrum bands (i.e., open/free spectrum), such as IEEE S-Band, IEEE L-Band, and ISM, fixedly defined in their hardware, under the same fixed spectrum allocation policy [31].

In this context, CR emerges as a promising technology to solve these issues by enabling DSA [10]. CR-based UAVs are able to select idle spectrum bands for communicating. Thus, the UAV overall communication quality increases when using CR, especially in overcrowded areas.

#### 4.1.4. Application Requirements

UAVs may often be deployed in missions where they are expected to broadcast live video and to send high definition pictures to the base station. However, live stream broadcast tolerate packet loss, but require a high bandwidth for the timely data delivery. Sending high definition pictures is the opposite operation, as the UAV may need a low bandwidth with no packet loss tolerance, but with delay tolerance [9].

While traditional UAVs may face this problem, CR-based UAVs may easily deal with it. CR-based UAVs may change their communication frequency accordingly to application requirements. Thus, a UAV equipped with CR technology may be live streaming video and switch to a low bandwidth to send a large file when required. This feature not only optimizes the overall network performance of CR-based UAVs, but it also opens new application opportunities for these aircraft. For instance, they can be used to improve the communication performance of terrestrial users in 5G, through the UAV-assisted 5G communications [32].

### 4.2. Potential Applications

In the last few years, emerged a growing trend to rely on UAVs as a support tool on critical missions, such as military surveillance [33], forest fire monitoring [34], crop monitoring [35,36,37], traffic surveillance [38], border patrolling [39], natural disasters area surveillance and support [40,41,42], and commercial drones [43]. However, these areas usually suffer from infrastructure issues that could possibly lead to spectrum scarcity or anomalous PUs behavior [44,45]. Thus, an integration between UAVs and CR could be useful for these scenarios due to its spectrum sensing and handover capabilities.

As well as the spectrum uncertainty scenario set up by emergency conditions, densely populated areas such as big cities can likewise go through overcrowded frequency bands, mainly motivated for the growing volume of wireless network users. Although spectrum scarcity is perceived as a harmless effect, the falling of a UAV in a crowded area due to an unstable communication could inflict much damage depending on the aircraft size and set up [46]. One potential advantage of CR utilization is the potential increase in the Quality of Service (QoS) caused by applying the spectrum handover capabilities to identify and relocate to less occupied frequency bands, thus adding to the stability of the data transmission between UAVs and their base stations.

In order to avoid the spectrum scarcity caused by densely populated areas, the UAV can previously adjust its flight path considering the spectrum availability, as applied in [47] using cellular data network. To accomplish this application, it can be done a mapping of several frequency bands of the area in which the UAV would be deployed, therefore enabling the occupancy pattern recognition and, subsequently, prediction by machine learning (ML) algorithms [48].

#### 4.2.1. Internet of Flying Things

The increased demand for highly connected devices originated the concept of IoT. In IoT, a massive number of “things” (i.e., devices) are connected to the Internet, in order to exchange information for the most diverse applications and purposes, from gains in efficiency to expansion of functions.

Because of the IoT rise, there is a major demand for integrating UAVs with this network. However, due to the special features involving Flying Ad hoc Networks (FANETS) and these aircraft, the IoFT was created. IoFT is a new research field that differs to IoT in scope of research, despite being related areas.

IoFT specific challenges range from regulatory issues to hardware and software limitations. Regarding security, as the UAVs are critical embedded systems, so a network security issue may generate a safety issue. The security challenges in IoFT are diverse, involving the physical, data link, transport, session, and application layers [7].

#### 4.2.2. UAV-Aided 5G

UAVs may play an important role for 5G and beyond 5G (B5G), as future dronecell networks [49]. Because cellular-connected UAV communication has unique characteristics related to ground conventional cellular communication, this field offers new research challenges and opportunities. Zeng et al. [5] presented an overview of 5G in UAV communications, as well as emerging technologies and potential challenges.

CR is an enabling technology for using UAVs in 5G networks. In 5G, CR-based UAVs may simultaneously access the wireless channel and fulfill their different roles in traffic surveillance, disaster management, and package delivery [32].

### 4.3. Hardware Characteristics

Usually, CR hardware consists of a radio platform, typically an SDR, and a computer platform. The Wireless Open-Access Research Platform (WARP) from Rice University [26] and the USRP from Ettus Research [25] are the most commonly deployed radio platforms for CR [27].

A recent implementation of SDR, however, has been carried out using various methods rather than considering the integration with UAVs. As a consequence, for UAVs, overhead, energy consumption, and time delays associated with conventional SDR are constraints. Alternatively, Young and Bostian [27] built the SKIRL, a simple and low-cost RFIC-based RF CR platform suitable for the experimentation of small radio-controlled UAVs. The Hope RF RFM22B [50] was used as the radio platform, whereas the BeagleBoard-xM [24] single board was deployed as the computer unit. This was done to reduce resource consumption and simplify CR design.

**Figure 4:**
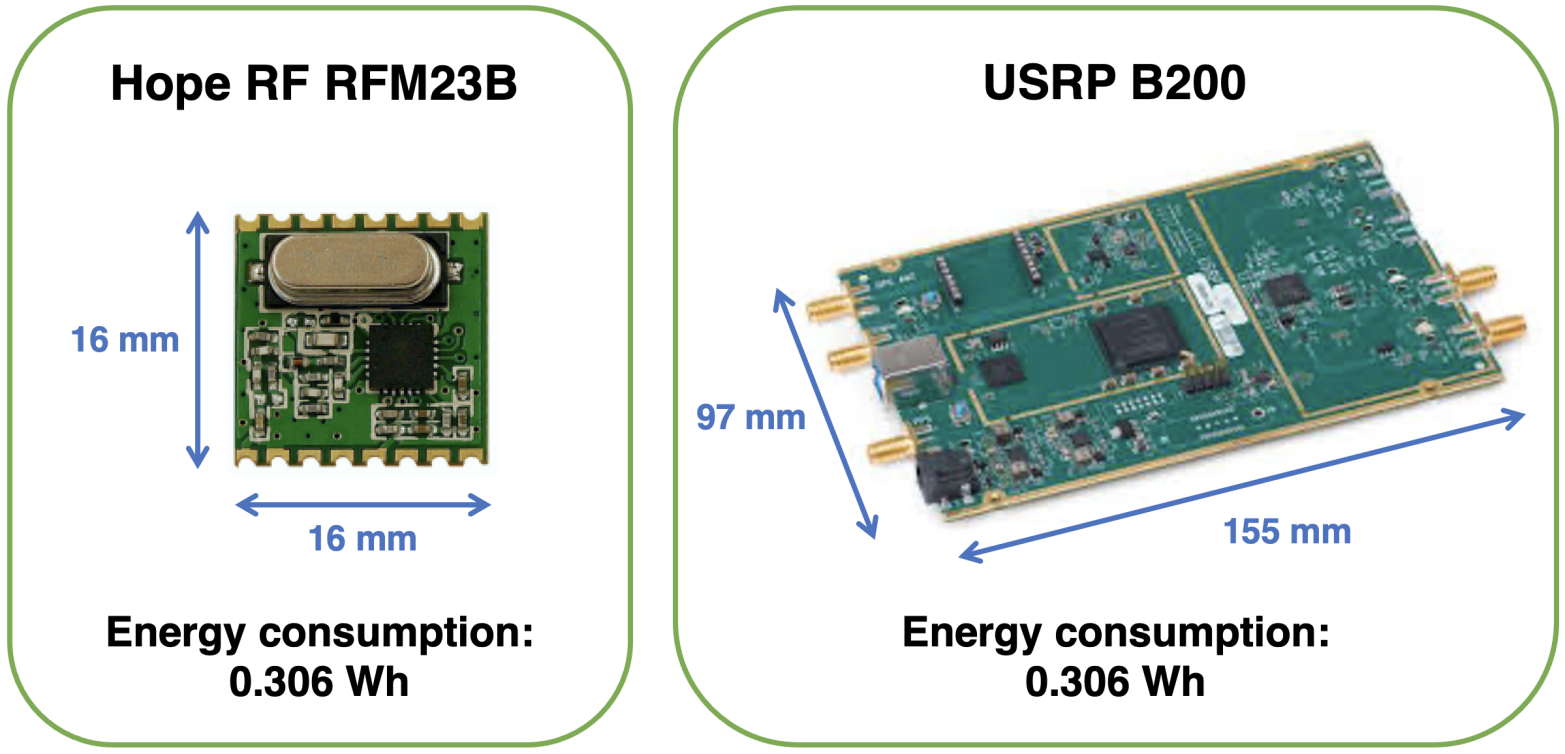
Physical aspects of the Hope RF RFM23B on the **left** and the USRP B200 on the **right**. The boards are shown in different size scales. Adapted from the work in [51].

Figure 4 shows the physical dimensions of the USRP B200, 97 × 155 millimeters, and the Hope RF RFM23B, 16 × 16 millimeters. For UAVs, particularly for micro UAVs, where physical space is limited, so if a large board is inserted to it, series of changes may be triggered in the aircraft system, configuration, and scale. Thus, this difference in size is critical. Moreover, the energy consumption of the Hope RF RFM22B is just 0.306 Wh in the worst case scenario. The USRP B200, on the other hand, may have up to 4.092 Wh of energy consumption. Because the energy capacity of a LiFe SourceHCAM6426 UAV lithium battery, a typical UAV battery, is just 12 Wh, energy consumption is a key aspect to take into account in this context.

### 4.4. Software Characteristics

The CR software design and applications can be divided into multiple categories, from MAC protocols and routing algorithms to machine learning approaches used to predict channel occupancy. In this work, we summarize three of those categories: (i) spectrum sensing algorithms, focusing mainly on transmitter detection and identification of spectrum holes [52,53]; (ii) methods to perform the spectrum handover [54]; and (iii) and simulation software [55].

#### 4.4.1. Spectrum Sensing

The literature commonly refers to spectrum sensing as the procedure of gathering and analyzing radio data to establish the spectrum occupancy [56]. In order to detect transmitters, some well-known methods can be employed, i.e., Energy detector, Matched-filter, and Cyclostationary feature detection. These algorithms are connected with two hypotheses formally defined as [57,58]

(1) $$x\left(t\right)=\left\{\begin{array}{cc} n(t), & H_{0}\\ s(t)+n(t), & H_{1} \end{array}\right.$$

where $H_{1}$ indicates the presence of a licensed user, whereas $H_{0}$ represents the null hypothesis. *x* represents the received signal, *t* is the time sample, and *s* and *n* denote the PU signal and the additive noise, respectively.

In cases where the signal receiver has no prior information about the PU signal, the energy detector algorithm may be deployed at a low computational cost. It functions by comparing a signal sample from the data to a predefined threshold, where a value higher than the threshold suggests the presence of a primary user, whereas a value smaller than the threshold implies the lack of band utilization. The main downside of this approach is the fixed threshold value, as noise power can differ over time possibly assuming unknown behavior. In this case, the threshold could be surpassed by the noise power, indicating an invalid PU presence in a given spectrum band [59,60].

The matched-filter contrasts the current signal with previous collected samples from the same transmitter, in comparison to the energy method. It appears to be more precise, has a shorter sensing time and maximizes the ratio of signal to noise (SNR). However, the need for prior knowledge of the form of the transmitter signal restricts its feasibility only to the point where licensed users cooperate [61].

In order to check whether or not the transmitted signal has periodicity, the cyclostationary feature detection adopts a spectral correlation function (SCF) [62]. Unlike the previous approaches, it helps the CR user to distinguish between noise and user signal, improving the efficiency of the algorithm in channels where there is greater noise [63]. Because of its computational complexity, perhaps the most serious drawback of this approach is the need for long processing time, an undesired characteristic for small energy consumption systems such as UAVs.

Finally, machine learning approaches have been adopted in the literature to enhance the detection of transmitters. The authors of [64] achieved an overall success rate of over 99.50% in predicting PU presence by using an Artificial Neural Network (ANN) at different SNR frequencies. Similarly, Zhang et al. [65] proposed a cooperative detection device combining the energy detector with ANNs, adding a basic ANN for each SU, and a base station responsible for final decision-making, referred to as the Fusion Center. Matinmikko et al. [66] proposed a new Fuzzy logic system to adapt each spectrum scenario to the most suitable transmitter detection algorithm. The reader may refer to the works in [67,68] for further information concerning spectrum sensing techniques.

#### 4.4.2. Spectrum Handover

The channel occupied by an unlicensed should ideally be vacant when a PU arrives. This is desirable in order to generate minimal interference to the primary user transmission. The literature generally refers to the process of hoping to another channels as spectrum handover. It involves distinct strategies with regards to its integration with spectrum sensing, such as non-handover, pure proactive, pure reactive, hybrid, and ML approaches, which incorporates the preceding [54].

In the non-handover strategy, the SU remains idle until the PU leaves the channel, resuming the transmission of data later. Although spectrum sensing is limited solely to current channel monitoring, the drawback of this concept is unveiled during a long PU transmission, which significantly restricts the transmission time available to the SU, in terms of detection of PU arrivals and departures.

Unlike non-handover, before and after the arrival of the PU, both pure proactive and reactive strategies concentrate on handover to an idle channel, respectively. The pure proactive algorithm tries to predict the arrival of the PU while perceiving the atmosphere to locate a spectrum hole based on the traffic pattern of the channel. On the contrary, after the identification of the PU, the pure reactive approach only senses and switches to an unused channel. A potential disadvantage in the pure proactive method may be created by a poor prediction of traffic, leading to an unnecessary handover, whereas the pure reactive approach may have a greater handover delay just after a PU arrival due to the execution of the spectrum sensing stage.

The hybrid handover strategy, taking advantage of the advantages of both methods, incorporates both the proactive spectrum sensing phase, perceiving the spectrum holes before the arrival of the PU, and reactive handover action. Therefore, due to the proactive process, the handover delay is reduced, and not every PU arrival needs to be predicted by the algorithm. However, the backup channel can become obsolete before use, as in the proactive method, thus driving the algorithm to perform a supplementary spectrum sensing phase.

The literature has stressed the utility of ML algorithms to overcome the complexities of spectrum handover. Trigui et al. [69] developed a method of negotiating multi-agent systems that enables SUs to migrate opportunistically to the most adequate spectrum band provided its characteristics, achieving around 97% of spectrum utilization. An investigation on the Hidden Markov Model (HMM) application for spectrum handover and simulated data showed that in detecting transmission opportunities, this technique can give SUs greater accuracy [70]. Finally, in order to achieve dynamic handover management, Anandakumar and Umamaheswari [71] suggested a supervised Machine Learning (ML) approach referred to as Spectrum Particle Swarm Optimization (SpecPSO), using Visitor Location Register and Home Location Register databases to train the algorithm.

#### 4.4.3. Simulation Tools

All levels of abstraction, from the physical layer to protocols and routing, should be associated with a full CR simulation program. We have not found a method in the literature that embodies all these characteristics. The only way to do this, to the best of our knowledge, is to combine open source resources, such as radio simulators (e.g., GNU Radio [72] and CogWave [73]) and general network simulators (e.g., Omnet++ [74] and ns-3 [75]).

GNU Radio is a free and open source software designed to use its graphical user interface flow graphs to simulate radio transmissions and signal processing (GNU Radio Companion). It could be compared to LabView [76] and Simulink [77]. Standard flow graph blocks encompass waveform generators, modulators, instrumentation sinks, math operators, filters, and Fourier analysis. It also facilitates the development of new blocks using the programming language C++, as well as the design of the flow graph using Python. In addition, GNU Radio can be connected to SDR hardware, thus allowing simulations from the testbed to be used.

Another open-source program suggested for designing CR waveforms is CogWave. It involves many modulation systems, including multichannel DAA-OFDM, Fathers, and others from GNU Radio (e.g., OFDM, BPSK, and QPSK). During run-time, CogWave is able to reconfigure the modulation scheme and can communicate with SDR hardware, as well as GNU Radio, to provide real-time transmission between USRP devices.

Omnet++ and INET [78] have been widely used in the literature to simulate CR networks for general network simulators [79,80,81]. Omnet++ offers a C++ component-based architecture where modules and components can be assembled using a graphical user interface or a high-level network description language, similar to flow graphs in GNU Radio (NED). Its modular architecture therefore eases the reusability of the built models. Furthermore, INET offers protocols, templates, routing, and mobility simulation as an open-source model library for Omnet++.

Another well-established tool is the ns-3. It is an open discrete-event simulation environment for network research that provides C++ libraries of models for wired protocols, IP and non-IP, wireless, dynamic routing protocols, and so forth. For instance, the ns-3 has been applied in the simulation of CR networks regarding spectrum handover [82], data collection [83], and channel sharing [84].

It is necessary to note that many programming languages can be used to build a custom simulator, as they provide access to several network and scientific libraries (e.g., Java, C++, Python, and Julia). However, most simulation scenarios encountered in the literature are complex enough to require thousands of lines of code, turning this option impractical for most of the problems.

### 4.5. Spectrum Mobility

When a PU resumes transmission through the same channel as the SU, the latter has to vacate the channel by suspending its transmission and restart communication through a vacant channel. This CR feature is called Spectrum Mobility [54].

It is considered a daunting problem due to the erratic behavior of the wireless medium in combination with the high mobility of UAVs. Thus, network protocols for ground-based networks could not perform as expected for UAVs, and their high mobility should be taken into account when designing CR-based UAV transport protocols [9,85].

One of the forms in which an SU does not trigger disruption in a PU-licensed band is to perform a spectrum handover as soon as the SU detects the existence of the PU. In general, UAVs take their places in a CR network as SUs, and they are likely to conduct a high number of spectrum handovers, as they will be on a mission throughout their route in the presence of various PUs. Therefore, spectrum handover is a key element in CR-based UAVs.

**Figure 5:**
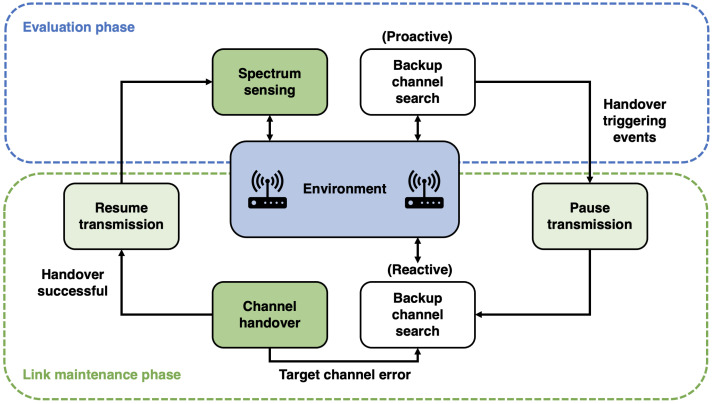
Spectrum handover process. Adapted from the work in [54].

A general spectrum handover process is shown in Figure 5. A SU keeps monitoring the spectrum environment during the evaluation phase by sensing it. The link maintenance process begins when a handover signal is identified, then the SU pauses its transmission and performs a channel handover to a backup channel and resumes its transmission, returning to the evaluation phase.

A handover trigger could be provided by a PU or by the SU itself, depending on the application purposes and signal quality. Depending on the spectrum handover strategy, a backup channel could be searched proactively during the assessment process, and a channel handover could also be performed proactively.

On the other hand, both backup channel scanning and channel handover are performed reactively in a reactive technique. A new backup channel has to be checked, and another channel handover attempt is made if a channel handover is not successful due to any incident affecting the target channel (e.g., a PU started a transmission through the target channel) [54].

Considerable energy consumption is required in a spectrum handover. Furthermore, some communication problems, such as packet loss and delay, may increase during the spectrum handover process. In a spectrum handover, delay is also a significant performance factor [86].

**Figure 6:**
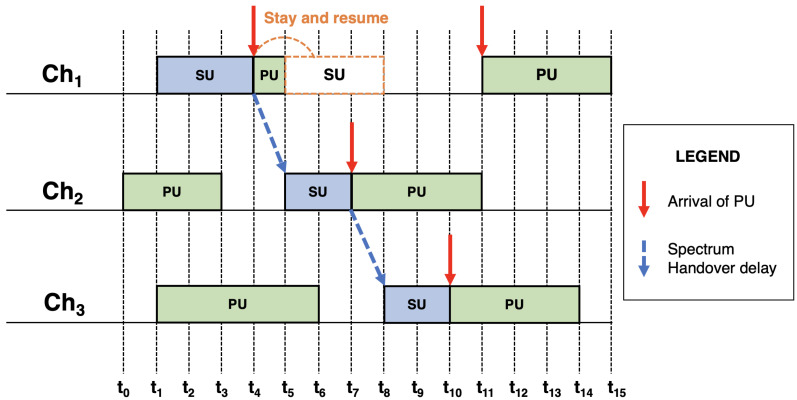
Spectrum handover delay caused to an SU. Adapted from the work in [87].

Figure 6 demonstrates the effect of the spectrum handover delay over a SU after many PU channel reclaims. In that case, an SU establishes a connection whose operation requires the completion of 7 time slots. However, due to successive PU arrivals, the process needs 9 time slots. Nevertheless, as the existence of a PU on channel Ch1 is quite short, the first SU channel handover was unnecessarily executed, which means that the SU was submitted to excessive channel handover costs [87].

UAVs designed for real-time multimedia broadcasting can cause unsatisfactory user experience, as spectrum handover latency can have a significant effect on delay-sensitive applications. For optimal transmission, therefore, delay reduction has to be considered [88].

## 5. Developing a Simple and Low-Cost CR-Based UAV Testbed

One of the main obstacles when integrating CR into UAVs is the energy consumption. Devices such as USRP B200 [25] have a consumption around 4.1 Wh, whereas a commercial quad-rotor UAV like DJI Phantom 4 [89] has a LiPo 4S battery with 81.3 Wh of capacity, which ensure a max flight time up to 28 min. This issue has been subject of empirical evaluations in Young and Bostian [27], where the authors apply the Hope’s RF RFM22B [50] to greatly reduce the energy utilization (0.3 Wh), although it likewise reduces the coverage range from USRP’s 70 MHz–6 GHz Hope’s 433–915 GHz.

The employment of miniaturized SDR devices could also help to reduce the additional weight load of UAV. For instance, Phantom 4 weights 1380 g, whereas USRP B200 weights 350 g. In comparison, Hope’s RF RFM22B weights only 1.8 g. The additional load could influence not solely the weight capacity of the aircraft, but also the energy consumption. Ultimately, the impact on the load capacity of the UAV poses as a major drawback on mobile manipulating UAVs [90].

Finally, there is no conclusive information referring to the maximum transmission distance of both USRP’s and Hope’s SDRs, as well as for other manufacturers. A limited transmission distance can be a tricky feature, potentially limiting the assimilation of CR into UAV Swarms. One probable solution to this shortcoming is the use of signal amplifiers; however, it is essential to note that additional weight load could result in less flight time, especially in swarms where the UAVs individual weight could reach 500 g in medium range devices or even less in short range devices.

### 5.1. The Testbed Components

The first step towards building this testbed is to develop and test a CR platform with the purpose to integrate it with the UAV. We developed one using a Raspberry Pi 3 connected to an RTL SDR 820T2 [91] as the radio front-end with a basic antenna. The RTL SDR 820T2 is a simple and low-cost USB SDR receptor, which frequency coverage varies from 30 MHz to 1.8 GHz.

Although the RTL SDR 820T2 does not support wireless data transmission (it only supports wireless data reception), its low-cost feature makes it a valuable tool for CR experiments, as most of the gaps are not found in the transmission itself, but in the received data processing and decision-making. For example, to evaluate a given spectrum handover algorithm, a CR device is not required to transmit wireless data itself. It only needs to sense the environment and decide whether to vacate the channel.

We choose a Parrot AR.Drone 2.0 [92] UAV to be integrated with the CR platform built for this work. Because Parrot AR.Drone 2.0 is a typical commercial drone widely used for entertainment, it fits the “keep it simple” requirement of this testbed, and it is also appropriated to test the CR platform suitability to mini-UAVs. Because the Parrot AR.Drone 2.0 is not open in hardware nor in software (except for an SDK intended for the development of smartphones, smartwatches, or VR glasses applications [93]), it is not possible to make any changes directly to the UAV. Therefore, here we use Parrot simply as the UAV platform; the Raspberry Pi 3 is used as the CR computer component. However, both the CR platform and the Parrot AR.Drone 2.0 run Linux operating systems [93]; therefore, this work results are equally valid not only for Linux-based computer platforms (like the Raspberry Pi 3), but also for open-source/open-hardware Linux-based UAVs. Note that for such UAVs it may be possible to use the same single board as the computational unit of both the CR and the UAV itself.

**Figure 7:**
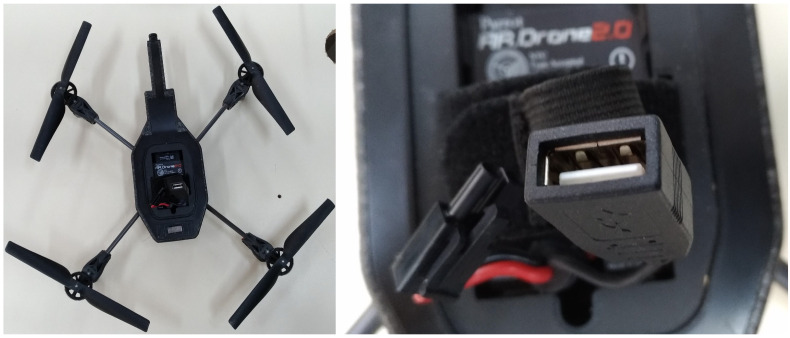
The Parrot AR.Drone 2.0 without its hull, highlighting its USB port.

Moreover, this UAV provides a USB port on its top. This port can be used as an interface to connect the CR platform to it. Figure 7 shows a picture taken in the laboratory of the Parrot AR.Drone 2.0 without its hull, in which it is possible to see its USB port.

Because of the low energy supply provided by the Parrot AR.Drone 2.0, it was necessary to efficiently adapt the CR platform. We changed its operational system to Ubuntu Mate, in order to have a better general compatibility with third-party software and more control over unnecessary background processing. It was also integrated with RLTSDR-Scanner, which is an open-source cross platform Python frequency scanning tool for RTL SDR [94].

**Figure 8:**
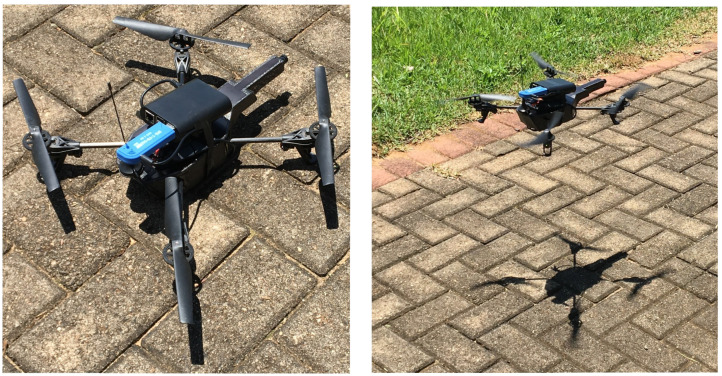
The CR-based UAV prototype developed for this work.

Finally, it was possible possible to deploy the CR-based UAV prototype outdoors. Figure 8 shows the prototype developed and deployed in this work. Because of the influence that an indoor environment could cause to radio data collection (for instance, because of walls, other devices, etc.), outdoor data collection is a key factor to determine the quality of the collected data.

### 5.2. Outdoor Radio Data Collection of a Jamming Attack

The experiment we conducted in this work comprises executing a typical jamming attack to the CR-based UAV prototype outdoor. The CR should detect the jammed frequency range as busy and then switch to a backup frequency, thus avoiding the jamming attack consequences.

In this experiment, we used a jammer device capable of jamming the frequency 432 MHz and the CR-based UAV testbed. First, we deployed the CR-based UAV sensing the 432 MHz frequency. Second, we used the jammer device to execute the jamming attack to that frequency. The CR-based UAV then sensed the high signal level in that frequency and it autonomously switched to its backup frequency. Although it was an outdoor experiment, it was important to isolate it, keeping it away from other variables. Therefore, we experimentally defined its backup channel as 440 MHz and its level threshold to −40 dB. It was necessary to avoid that other PUs and/or SUs interfered in the experiment, and to reduce the incidence of false alarms.

**Figure 9:**
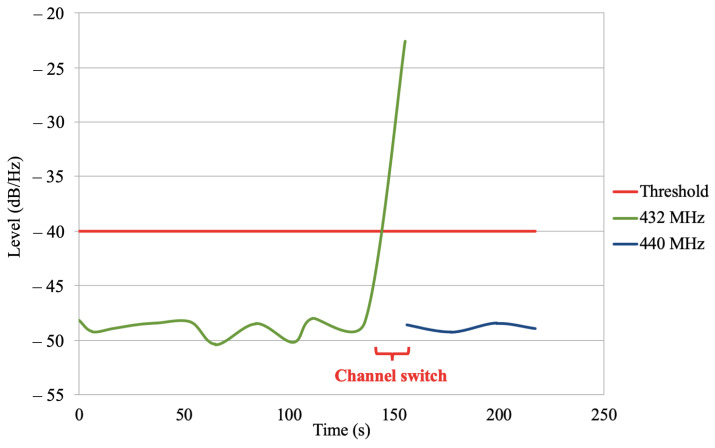
Data collected using the CR-based UAV. It kept using the 432 MHz frequency until it sensed a transmission level over the threshold. It then switched to its backup channel at 440 MHz.

Figure 9 shows that the CR-based UAV prototype performed as expected under a jamming attack.

**Figure 10:**
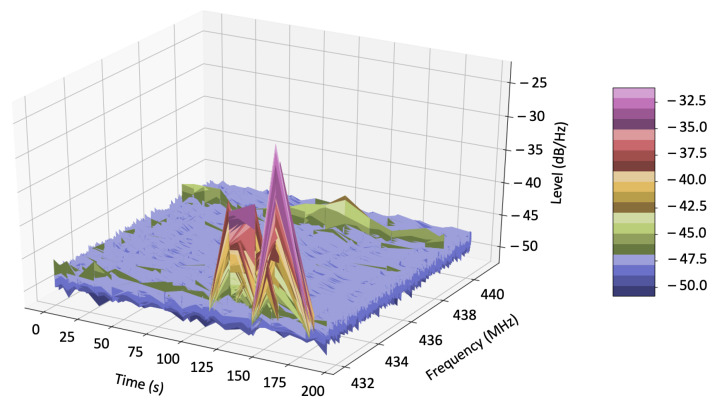
Data collected using the CR platform. It shows a map of the level in dB measured within the frequency range from 432 MHz to 441 MHz throughout the time.

For a clearer understanding of the radio spectrum environment within the frequency range from 432 MHz to 441 MHz, we used the CR platform to make a data collection during a jamming attack, in similar circumstances to the CR-based UAV testbed experiment. Figure 10 shows the data collected. It highlights the 432 MHz frequency level discrepancy under the jamming attack, related to other frequencies within the measured range.

These results show that even switching to a channel around the same frequency bands could be enough to avoid a jamming attack, considering the attacker does not have access to the new channel. Furthermore, note that we used a simple reactive algorithm based on static channel noise threshold, but especially proactive algorithms may benefit from such data collections, as Machine Learning techniques could be trained and tested over real-scenario data.

## 6. State-of-the-Art of CR-Based UAVs

We conducted a review in November 2020 in search of works involving UAVs and SDR and/or CR. The main idea was to find the state-of-the-art of CR-based UAVs. As SDR-based UAVs represent somehow a step backwards for CR-based UAVs, they were also included in this review.

We evaluated a total of 520 publications from four different digital libraries. However, the majority of these articles were identified marginally related to the scope of this work. Thus, the final studies dataset obtained after analyzing them regarding the following exclusion criteria consisted of 64 articles:

1. non-English publications. Although we used English language keywords, we found a few publications in other languages. We had to exclude them merely because we would not be able to analyze them in depth;
2. papers that are not downloadable online;
3. publications that are not related to CR, SDR, or UAVs as defined in this work (other research fields);
4. papers focusing on CR or SDR applications other than UAVs wireless communication.

**Figure 11:**
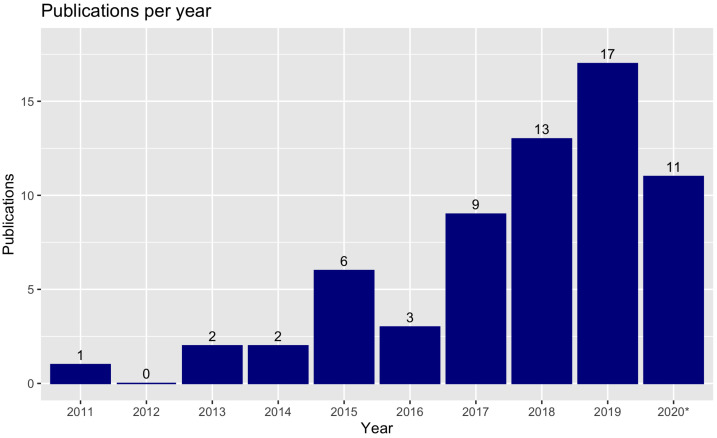
Related publications per year. The year of 2020 was evaluated from January until November.

Figure 11 outlines the number of publications per year, among those selected in this review. It shows an increasing interest over the recent years in this research field. Although the year of 2020 was evaluated from January until November, it already has a high number of publications. Note that a number of papers might have been published from January 2020 to November 2020, but were not yet available online.

**Figure 12:**
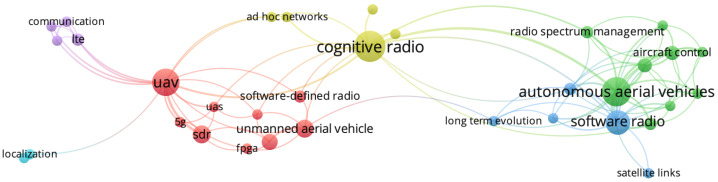
Keywords graph and occurrence mapping for all 64 articles selected on this review.

Figure 12 presents keywords graph and occurrence mapping for all 64 articles selected on this review. We consider keywords with at least two occurrences and at least one link to other keywords. Thereby, the keyword mapping consists of 34 keywords in total. In Figure 12, it is possible to note an expressive amount of links correlating UAV-related keywords and SDR-related keywords. However, although CR-related keywords are present in Figure 12, there are few links correlating them to UAVs. Thus, Figure 12 outlines the paucity of studies on the integration of CR and UAVs.

In order to measure how fit each selected publication is to CR-based UAVs research field, we analyzed each paper regarding the following aspects:

1. the work presents a theoretical analysis (Q1);
2. the work presents practical results (Q2);
3. the work involves a real SDR-based UAV (Q3);
4. the work is centered on CR-based UAVs (Q4);
5. the work involves a real CR-based UAV (Q5).

For each publication, each aspect is analyzed, and it is evaluated as “yes”, if the aspect is present in the paper, or “no” otherwise. Table 2 presents all selected publications in this review, as well as their publication year and aspects analysis.

In general, these works can be separated in two groups: one using SDR-based UAVs for different applications, and another studying the impact of integrating CR technology with UAVs, mainly with theoretical analysis and simulation results.

A notable exception to this is Sklivanitis et al. [119]. The authors designed, developed, and validated through real indoor and outdoor experiments a CR platform for UAVs. Their experiment involves on-ground CR nodes interacting with a CR-based UAV, and it shows that their proposed platform optimizes wireless networks subject to interference, thus maximizing their throughput. To the best of our knowledge, their work presents the first CR-based UAV real experiment in the literature.

## 7. Challenges and Future Research Directions

**Figure 13:**
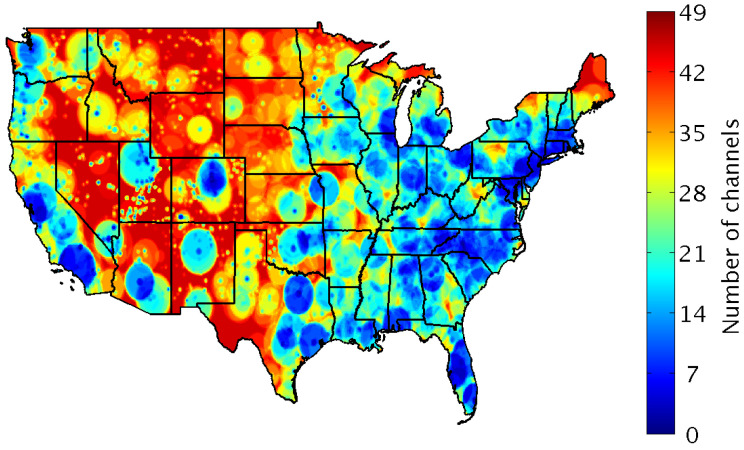
Amount of 6 MHz TV channels available for SUs in the United States in 2015. Adapted from the work in [90].

To better grasp how the occupation pattern behaves in spectrum bands, a channel white-space mapping may be helpful. As the authors of [90] demonstrated, distinct areas on the map ( Figure 13) may have a varied spectrum behavior. Therefore, a data exploration study like this is essential to improve the PU arrival prediction and, consequently, enhance the spectrum handover. In addition, a white-space mapped area may be crucial in order to achieve an UAV flight path guided by spectrum occupancy.

Although some studies [153,154,155,156] have successfully employed Machine Learning algorithms to predict the PUs arrivals, most of them use simulated data. Even though simulated data have importance in proof-testing new approaches, real data are indispensable when integrating UAVs to CR. Likewise, these studies generally concentrate on prediction of one or few channels, although a large range of channels may be preferable on UAV context.

As noted before, the proportion of SDR devices presents as a major challenge. Although devices such as Hope’s RF RFM22B may initially comply with this requirement, a device with a wider frequency range may be desirable for the use in UAV communication. In consonance with hardware improvements, to the extent of our knowledge, there has been no study concerning the software adjustments required when combining SDR and UAVs.

## 8. Conclusions

In this paper, we presented an overview on CR-based UAVs, as well as their hardware and software requirements. We also indicated the first steps taken towards integrating CR with UAVs, and a review of the state-of-the-art of CR for UAV communications. This work has also shown that several questions remain unclear, in particular regarding the impact of CR to UAVs energy consumption, and the effects of UAVs mobility to CR spectrum mobility and spectrum sharing. We also specified steps to build a simple and low-cost CR-based UAV testbed.

Future work shall consider a channel occupation mapping aiming at achieving an UAV flight path guided by spectrum occupancy. PU arrivals prediction based on Machine Learning algorithms on real data collected by UAVs is a key element concerning the study of CR algorithms for UAVs. Finally, CR hardware and software that meet the constraints and requirements of UAVs are also needed.

## Figures and Tables

**Table 1:** Unmanned aerial vehicles (UAVs) classification. Adapted from the work in [9].

UAV	Weight (kg)	Altitude (km)	Endurance (h)
Micro	0.1	0.25	1
Mini	<30	0.15–0.3	<2
Short range	200	3	2–4
Medium range	150–500	3–5	30–70
Long range	-	5	6–13
Endurance	500–1500	5–8	12–24
Medium altitude,	1000–1500	5–8	24–48
long endurance			
High altitude	2500–12,500	15–50	24–48
long endurance			

**Table 2:** Reviewed publications and their explored topics. On the columns Q1 to Q5, cells filled in gray color represent “yes” for the respective column, and “no” otherwise.

Publication	Q1	Q2	Q3	Q4	Q5
Chen et al. [95]					
Young and Bostian [27]					
Miko and Nemeth [96]					
Harounabadi et al. [97]					
Brown et al. [98]					
Reyes et al. [10]					
Guevara et al. [99]					
Anderson et al. [100]					
Mikó and Németh [101]					
Andryeyev et al. [102]					
Saleem et al. [9]					
VonEhr et al. [103]					
Jacob et al. [15]					
Tato et al. [104]					
Murphy et al. [105]					
Sboui et al. [106]					
Gutierrez et al. [107]					
Gonzalez and Fung [108]					
Horapong et al. [109]					
Zhang et al. [110]					
Ghazzai et al. [111]					
Cai et al. [112]					
Noble et al. [113]					
Petrolo et al. [114]					
Shi et al. [115]					
Huang et al. [116]					
Shi et al. [117]					
Liu et al. [118]					
Sklivanitis et al. [119]					
Pärlin et al. [120]					
Dunne and Keenlance [121]					
Shamaei et al. [122]					
Santana et al. [51]					
Jadon et al. [123]					
Torabi et al. [124]					
Xu et al. [125]					
Pan et al. [126]					
Shen et al. [127]					
Aftab et al. [128]					
Adane [129]					
Almasoud and Kamal [130]					
Matheou et al. [131]					
Wang et al. [132]					
Murphy et al. [133]					
Hasan et al. [134]					
Che et al. [135]					
Nie et al. [136]					
Radišić et al. [137]					
u. Hasan et al. [138]					
Yuheng et al. [139]					
D’Alterio et al. [140]					
Zhao et al. [141]					
Mohanti et al. [142]					
Liang et al. [143]					
Kornprobst et al. [144]					
Bertizzolo et al. [145]					
Figueira et al. [146]					
Hasan et al. [147]					
Powell et al. [14]					
AbdulCareem et al. [148]					
Zambrano et al. [149]					
Sommer et al. [150]					
Liu et al. [151]					
Krayani et al. [152]					

## Data Availability

The data collected by the CR-based UAV testbed are available online: https://github.com/GMDSantana/pub-files/tree/main/sensors-2021.

## Abbreviations

The following abbreviations are used in this manuscript:

AERPAW	Aerial Experimentation and Research Platform for Advanced Wireless
ANN	Artificial Neural Network
B5G	beyond 5G
CR	Cognitive Radio
DSA	Dynamic Spectrum Access
ETSI	European Telecommunications Standards Institute
FANETs	Flying Ad hoc Networks
GNSS	Global Navigation Satellite System
GPS	Global Positioning System
HMM	Hidden Markov Model
IoFT	Internet of Flying Things
IoT	Internet of Things
ITU	International Telecommunications Union
MAC	Medium Access Control
ML	Machine Learning
PU	Primary User
QoS	Quality of Service
SCF	Spectral Correlation Function
SDR	Software-Defined Radio
SNR	Ratio of Signal to Noise
SpecPSO	Spectrum Particle Swarm Optimization
SU	Secondary User
UAS	Unmanned Aerial Systems
UAV	Unmanned Aerial Vehicle
URLLC	Ultra-Reliable and Low Latency
USRP	Universal Software Radio Peripheral
WARP	The Wireless Open-Access Research Platform
Wi-FI	Wireless Network
